# A Case of Multiple Intratumoral Hemorrhage and Rupture of Hepatocellular Carcinoma after Starting Lenvatinib

**DOI:** 10.1155/2020/6659388

**Published:** 2020-12-19

**Authors:** Katsutoshi Ishihara

**Affiliations:** Department of Radiology, Yuai Memorial Hospital, 707 Higashiushigaya, Koga, Ibaraki, Japan

## Abstract

A man in his 80s was administered lenvatinib to treat multiple hepatocellular carcinomas. After starting lenvatinib, he was admitted to our hospital for fever and epigastric tenderness. Conjunctival icterus and conjunctival pallor were observed. Computed tomography showed high density areas in his tumors which were suggestive of intratumoral hemorrhage and tumor rupture. As a result, the patient underwent an emergent angiography and arterial embolization.

## 1. Introduction

Lenvatinib is a multityrosine kinase receptor inhibitor that inhibits vascular endothelial growth factor (VEGF) receptor [1]. Lenvatinib can be used to treat patients with unresectable hepatocellular carcinoma (HCC).

The most common treatment-related adverse events among patients who received lenvatinib included hypertension, diarrhea, decreased appetite, and weight loss [2]. In a phase II trial, one patient died of liver tumor rupture that occurred within 30 days after receiving the last dose of lenvatinib [3]. The present study describe the case of a patient with advanced HCC who experienced multiple intratumoral hemorrhage and concomitant tumor rupture after the onset of treatment with lenvatinib.

## 2. Case Presentation

**Figure 1:**
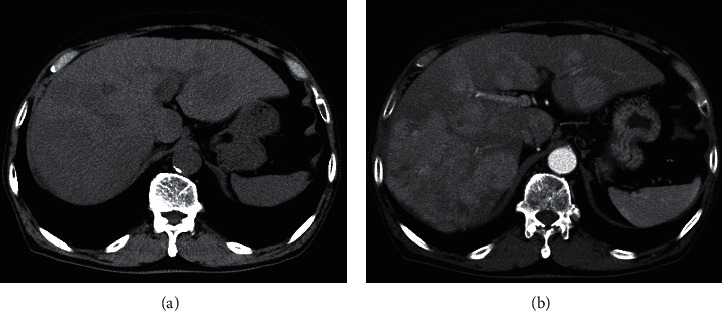
Nonenhanced computed tomography (a) and arterial phase of CT (b). Multiple liver tumors are depicted as low attenuation areas in nonenhanced CT and exhibited early enhancement in the arterial phase. These tumors were considered hepatocellular carcinomas.

A man in his 80s with liver cirrhosis due to hepatitis C virus referred to our hospital for further examination and treatment of multiple hepatic masses. Computed tomography (CT) revealed multiple HCCs in both lobes of the liver (Figures 1(a) and 1(b)). PIVKA-II was 1000.51 mAU/ml on admission. Lenvatinib was started at a dose of 8 mg/day because surgery and radiofrequency ablation was not feasible.

**Figure 2:**
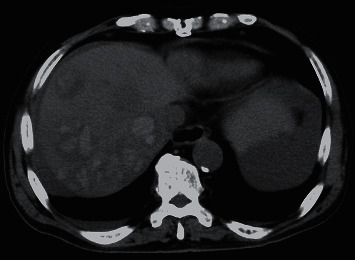
Nonenhanced computed tomography image. High-attenuation areas were observed in multiple HCCs considered to indicate multiple intratumoral hemorrhages.

**Figure 3:**
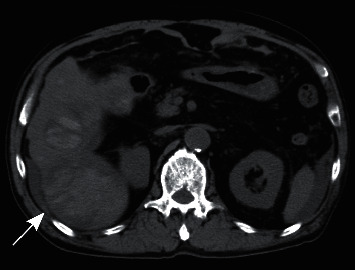
Nonenhanced computed tomography image. Perilesional blood was also observed near the hepatic tumor in the right lobe of the liver that was considered to indicate rupture of the HCC (arrow).

**Figure 4:**
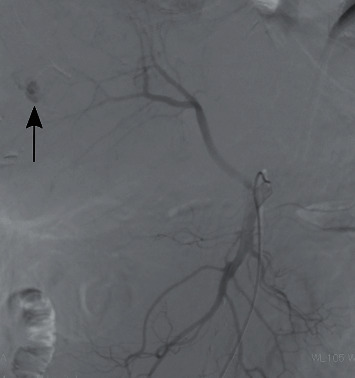
Pooling of contrast medium was observed on angiography (arrow). Right hepatic artery was replaced on the superior mesenteric artery.

**Figure 5:**
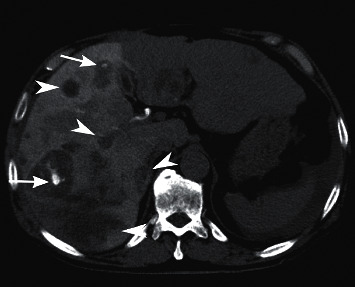
Arterial phase of computed tomography during right hepatic arteriography. Pooling of contrast medium was observed (arrows). Contrast effect of tumors was low (arrowheads) and it was considered to indicate decrease in vascularity of hepatocellular carcinomas.

**Figure 6:**
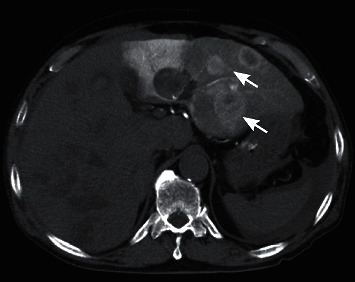
Arterial phase of computed tomography during left hepatic arteriography. In the left lobe of the liver, tumor stains were observed in more tumors than in the right lobe (arrowhead).

Six days after starting lenvatinib, the patient experienced some epigastric discomfort. However, no clinically significant findings in the gastrointestinal endoscopic examination were observed. Twelve days after starting lenvatinib, he was admitted to our hospital for fever and epigastric tenderness. Conjunctival icterus and conjunctival pallor were observed. Laboratory results on admission showed a hemoglobin value of 8.5 g/dL, white blood cell count of 16100/*μ*l, total bilirubin of 1.9 mg/dL, elevated transaminase (aspartate aminotransferase (AST) of 119 U/L; alanine aminotransferase (ALT) of 87 U/L), lactate dehydrogenase (LDH) of 406 U/L, and C-reactive protein (CRP) of 12.07 mg/dL. Platelet count was within normal limits. CT examination showed high-attenuation areas in the multiple HCCs (Figure 2), and perilesional blood was also observed near the hepatic tumor in the right lobe of the liver (Figure 3). Therefore, multiple intratumoral hemorrhage and HCC rupture were considered. The patient underwent an emergency angiography and arterial embolization. Pooling of contrast medium concerning intratumoral hemorrhage was observed in some tumors on angiography and CT during hepatic arteriography (CTHA) (Figures 4 and 5). CTHA also demonstrated a decrease in vascularity of the hepatic tumors mainly in the right lobe of the liver which was suggestive of a decrease in the viability of the tumor (Figure 5). In the left lobe of the liver, tumor stains were observed in more tumors than in the right lobe (Figure 6). Hepatic artery embolization was done with a gelatin sponge. Emulsion of lipiodol (2 ml) and epirubicin hydrochloride (20 mg) was also given before embolization to have an antitumor effect in the left lobe of the liver because tumor stains were observed in more tumors in the left lobe on CTHA than on the right lobe.

Eleven days after emergency embolotherapy, AST, ALT, and total bilirubin were recovered to normal levels. The hemoglobin level was recovered to 10.2 g/dl after embolization, and he was discharged from the hospital 18 days after embolotherapy. There was no fever or abdominal pain after discharge from the hospital. Administration of lenvatinib (4 mg/day) was resumed about two months after HCC rupture and intratumoral hemorrhage.

PIVKA-II was 1290.3 mAU/ml at three days after emergency angiography and 127.91 at two months after embolotherapy. Alpha-fetoprotein was within normal limits during the observation period.

## 3. Discussion

Lenvatinib is a multityrosine kinase inhibitor and inhibits tumor angiogenesis [1]. In the present case, some HCCs had decreased vascularity on CTHA. I believe that this decrease might be due to lenvatinib-induced inhibitions of tumor angiogenesis.

Sorafenib, like lenvatinib, is a multikinase inhibitor that inhibits VEGF. Rombolà et al. reported a case of HCC rupture and attributed the risk to sorafenib use [4]. Sorafenib inhibits VEGF signaling and is known to increase the risk of bleeding. VEGF is important for the survival of endothelial cells and for maintaining the architecture and integrity of the vasculature. Therefore, inhibition of the VEGF pathway may decrease the regeneration potential of damaged endothelial cells and may consequently increase the risk of hemorrhage [5]. The occurrence of hemorrhagic complications with lenvatinib use has been documented. In the REFLECT trial, cerebral hemorrhage was observed in 3 out of 462 patients [2]. In another phase II trial, one patient died because of a ruptured liver tumor within 30 days after receiving the last dose of lenvatinib [3]. Moreover, hemorrhagic events occurred in 35% of the patients treated with lenvatinib versus 18% of patients who received placebo against thyroid cancer [6]. In the present study, intratumoral hemorrhage and concomitant HCC rupture were observed. These phenomena may be attributable to the effect of lenvatinib as a VEGF inhibitor. Rupture of HCC may occur with the use of both lenvatinib and sorafenib.

High attenuation areas were seen in the multiple HCCs, and perilesional blood was observed near the hepatic tumor in the right lobe of the liver by CT examination in the present case. Therefore, multiple intratumoral hemorrhage and HCC rupture were considered. The pooling of contrast medium for intratumoral hemorrhage was observed in some tumors on angiography and CTHA. Uchida-Kobayashi et al. described that five of 68 cases treated with lenvatinib developed intraperitoneal or intratumoral hemorrhage, and they also observed pooling of contrast medium on angiography after intratumoral hemorrhage [7]. As they have pointed out, we also thought that pooling of contrast medium during angiography was similar to vascular lake phenomenon that was observed during TACE with drug-eluting beads (DEB-TACE). The etiology of the vascular lake phenomenon during DEB-TACE is not yet well understood. Seki et al. hypothesized that vascular lake phenomenon emerging during DEB-TACE could arise through the rupture of some of the vulnerable tumor microvasculature formed by tumor angiogenesis [8]. In our case, we thought that lenvatinib inhibited tumor angiogenesis and the remaining poorly developed tumor microvasculature tended to collapse. Therefore, pooling of contrast medium might be occurred and it suggested intratumoral hemorrhage.

The mechanism of spontaneous HCC rupture has not been understood. Several hypotheses have been proposed to explain the HCC rupture. It is said that tumor location is associated with spontaneous rupture of HCC [9]. Caudate lobe HCC or HCC which is subcapsular in location tends to rupture compared to a centrally located HCC because normal hepatic parenchyma surrounding the HCC can protect the tumor from rupture. Li et al. reported that most ruptured HCCs were localized to the left lateral segment (seg II and seg III) and right posterior-inferior segment (seg VI) [9]. These segments have relatively small room for a space occupying lesion compared to the other segments. Therefore, when the tumor grows beyond its capacity, the inner pressure splits open the surrounding parenchyma and tears the capsule leading to rupture. In the present case, ruptured HCC was localized to the subcapsular region in posterior-inferior segment. It might had been more prone to rupture because of the location of HCC and use of VEGF inhibitor.

There may not be a correlation between the dose of lenvatinib and hemorrhage because this report described that initiation doses of lenvatinib among the patients with hemorrhage were 12, 8, and 4 mg/day [7]. This report also described that the patients with hemorrhage had larger tumor than the patients without hemorrhage. So TACE prior to administration of lenvatinib for large HCC may be an option for precaution of HCC rupture.

This report describes multiple intratumoral hemorrhage and HCC rupture caused by lenvatinib administration in a patient with multiple HCC. It is important to know that HCC may be prone to rupture depending on the location of HCC and the use of lenvatinib.

## Data Availability

The data used to support the findings of this study are included within the article.
